# Myocardial perfusion cardiovascular magnetic resonance: optimized dual sequence and reconstruction for quantification

**DOI:** 10.1186/s12968-017-0355-5

**Published:** 2017-04-07

**Authors:** Peter Kellman, Michael S. Hansen, Sonia Nielles-Vallespin, Jannike Nickander, Raquel Themudo, Martin Ugander, Hui Xue

**Affiliations:** 1 National Heart, Lung, and Blood Institute, National Institutes of Health, DHHS, 10 Center Drive MSC-1061, Bethesda, MD 20892 USA; 2 Department of Clinical Physiology, Karolinska Institutet and Karolinska University Hospital, Stockholm, Sweden

**Keywords:** Myocardial perfusion, Flow, Perfusion quantification, Arterial input function, Gadolinium

## Abstract

**Background:**

Quantification of myocardial blood flow requires knowledge of the amount of contrast agent in the myocardial tissue and the arterial input function (AIF) driving the delivery of this contrast agent. Accurate quantification is challenged by the lack of linearity between the measured signal and contrast agent concentration. This work characterizes sources of non-linearity and presents a systematic approach to accurate measurements of contrast agent concentration in both blood and myocardium.

**Methods:**

A dual sequence approach with separate pulse sequences for AIF and myocardial tissue allowed separate optimization of parameters for blood and myocardium. A systems approach to the overall design was taken to achieve linearity between signal and contrast agent concentration. Conversion of signal intensity values to contrast agent concentration was achieved through a combination of surface coil sensitivity correction, Bloch simulation based look-up table correction, and in the case of the AIF measurement, correction of T2* losses. Validation of signal correction was performed in phantoms, and values for peak AIF concentration and myocardial flow are provided for 29 normal subjects for rest and adenosine stress.

**Results:**

For phantoms, the measured fits were within 5% for both AIF and myocardium. In healthy volunteers the peak [Gd] was 3.5 ± 1.2 for stress and 4.4 ± 1.2 mmol/L for rest. The T2* in the left ventricle blood pool at peak AIF was approximately 10 ms. The peak-to-valley ratio was 5.6 for the raw signal intensities without correction, and was 8.3 for the look-up-table (LUT) corrected AIF which represents approximately 48% correction. Without T2* correction the myocardial blood flow estimates are overestimated by approximately 10%. The signal-to-noise ratio of the myocardial signal at peak enhancement (1.5 T) was 17.7 ± 6.6 at stress and the peak [Gd] was 0.49 ± 0.15 mmol/L. The estimated perfusion flow was 3.9 ± 0.38 and 1.03 ± 0.19 ml/min/g using the BTEX model and 3.4 ± 0.39 and 0.95 ± 0.16 using a Fermi model, for stress and rest, respectively.

**Conclusions:**

A dual sequence for myocardial perfusion cardiovascular magnetic resonance and AIF measurement has been optimized for quantification of myocardial blood flow. A validation in phantoms was performed to confirm that the signal conversion to gadolinium concentration was linear. The proposed sequence was integrated with a fully automatic in-line solution for pixel-wise mapping of myocardial blood flow and evaluated in adenosine stress and rest studies on *N* = 29 normal healthy subjects. Reliable perfusion mapping was demonstrated and produced estimates with low variability.

**Electronic supplementary material:**

The online version of this article (doi:10.1186/s12968-017-0355-5) contains supplementary material, which is available to authorized users.

## Background

Myocardial perfusion can be evaluated with dynamic cardiovascular magnetic resonance (CMR) during the passage of a bolus of contrast agent. Most commonly, perfusion CMR is evaluated qualitatively, but objective quantitative evaluation would be more desirable. The potential benefits of quantification are: objective assessment, simpler and faster analysis, and the ability to detect disease with a global reduction in flow such as multi-vessel obstructive disease or microvascular disease. Quantification of myocardial blood flow using CMR was first proposed over 20 years ago [1, 2], yet qualitative interpretation of images remains the primary means available to clinicians. The desired output of a quantitative perfusion study is a map of myocardial blood flow in units of ml/g/min.

Quantification requires knowledge of the amount of contrast agent in the myocardial tissue and the arterial input function (AIF) driving the delivery of this contrast agent. Accurate quantification is challenged by the lack of linearity between the measured signal and contrast agent concentration. Ideally these measurements would consist of input and response curves in units of contrast agent concentration. Current, commercially available, myocardial perfusion sequences have not been optimized to yield accurate concentration curves and the observed signal intensity curves are not linearly related to the concentrations of contrast agent, i.e., there is a non-linear relationship between signal intensity and contrast agent concentration, which leads to quantification biases.

The main sources of non-linearity and bias are: spatial signal variations caused by the sensitivity profiles of the surface coils, imperfect saturation of magnetization during contrast bolus passage, T2* decay (and signal loss) caused by high contrast agent concentrations in the blood pool, and the non-linear signal response inherent due to saturation recovery that depends on the parameters of the imaging protocol. It has been proposed that some of the non-linearity of the AIF response curve can be mitigated by imaging the AIF during a separate injection of a bolus with lower concentration (the dual bolus approach)[3], but this approach has some practical drawbacks as it requires multiple injections and acquisitions. Moreover, there are other potential bias sources with his approach, since changes in breathing, etc. between the two measurements may introduce new sources of variation. Consequently, it is desirable to acquire the AIF curve simultaneously with the tissue response curve.

A dual sequence [4] approach, which separately optimizes the imaging protocols for blood and myocardium has been proposed. This approach may be more easily incorporated into a clinical workflow. The proposed dual sequence was optimized for perfusion quantification and was evaluated using a recently developed fully automatic in-line solution for pixel-wise mapping of myocardial blood flow [5]. This work characterizes the sources of non-linearity and presents a systematic approach to accurate measurements of contrast agent concentration in both blood and tissue of interest.

## Methods

### Sequence

**Fig. 1:**
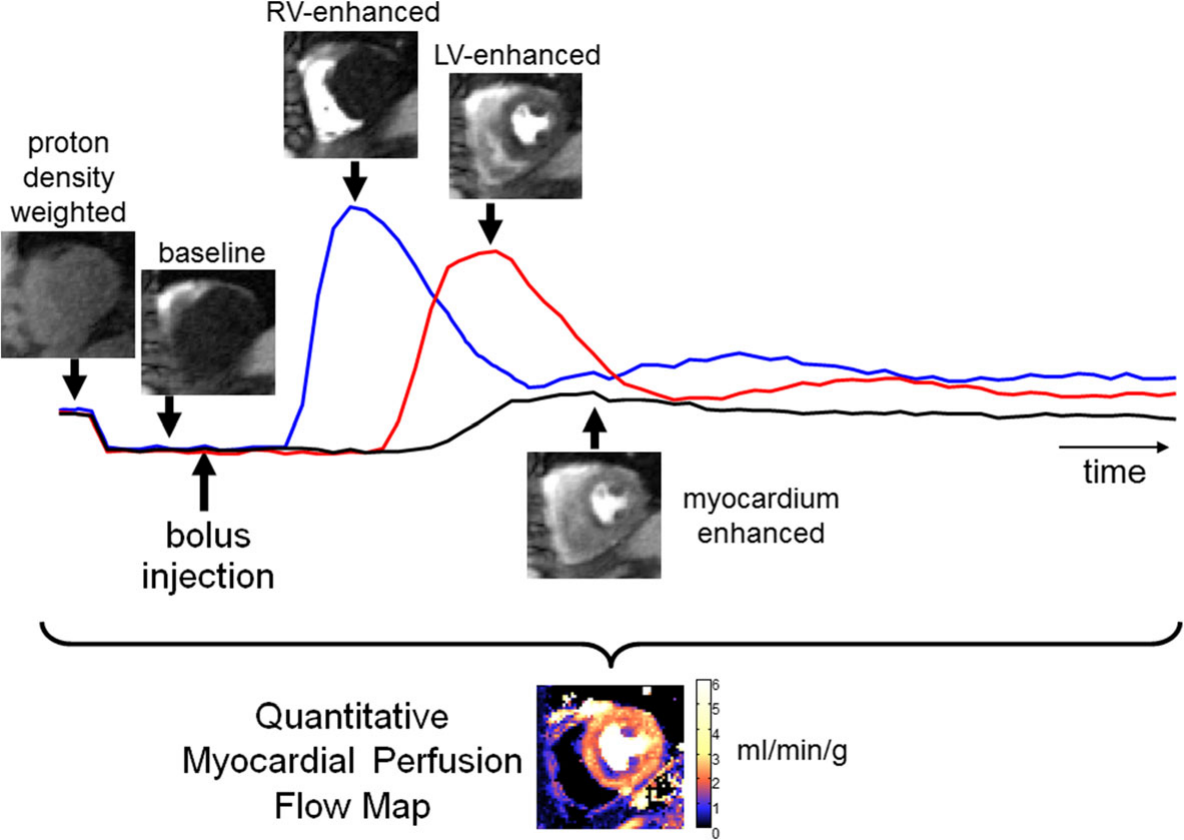
Illustration of first-pass contrast enhanced myocardial perfusion imaging showing different phases of image contrast during passage of the bolus for a subject with single vessel disease. Proton density weighted images are acquired at the start of acquisition prior to administering the contrast agent bolus. The complete time series of images are automatically processed to estimate pixel-wise myocardial blood flow maps which show regions of low flow in different color than normal flow, thereby reducing the time required to analyze the raw images. The time intensity signals represent the intensities of RV blood pool (*blue*), LV blood pool (*red*), and myocardium (*black*) regions. Note that flow map values are only valid for myocardium tissue and not blood pool regions or in non-tissue

A saturation recovery (SR) sequence was used for myocardial perfusion imaging during the passage of a bolus of gadolinium based contrast agent as depicted in Fig. 1 which is illustrated for a subject with single vessel disease. Baseline images were acquired prior to bolus administration and continued through the first pass. Typically, images were acquired for 60-90 heartbeats depending on the cardiac output. Proton density (PD) weighted images were acquired at the start.

**Fig. 2:**
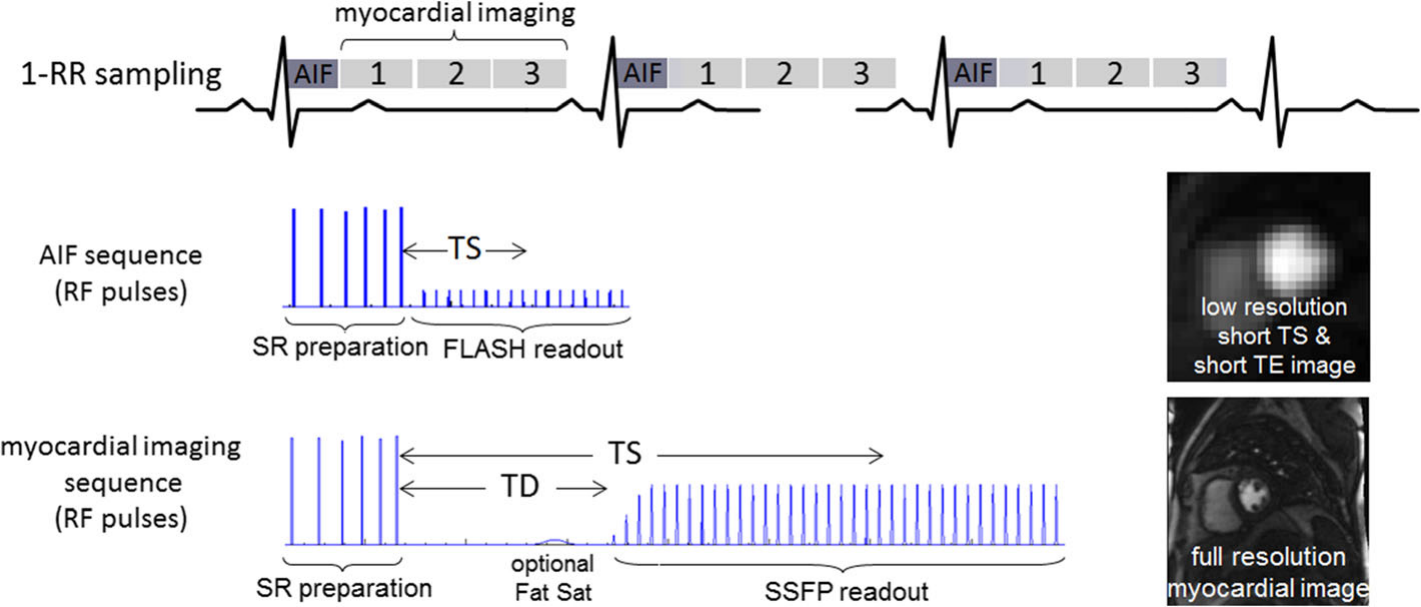
Overview diagram of “dual”-sequence for multi-slice 2D saturation recovery. The low resolution AIF image is acquired at the R-wave triggered followed by multiple full resolution myocardial perfusion images. Each image has a saturation recovery RF preparation consisting of a sequence of RF pulses and gradient spoilers followed by single shot image readout. The myocardial perfusion images have an optional chemical shift fat saturation. The AIF uses a FLASH readout, whereas the full resolution myocardial images may be either b-SSFP or FLASH. The AIF is acquired for a single slice every RR interval, whereas the myocardial perfusion images may be sampled every RR or every other RR interval to increase the overall number of slices. TD and TS are the trigger delay and saturation time, respectively

A multi-slice 2D SR dual imaging sequence is diagrammed in Fig. 2. Low resolution blood pool images used for estimating the AIF were acquired every heartbeat immediately following the R-wave trigger. Higher resolution images were acquired following the AIF and may be sampled every RR or every second RR if greater spatial coverage is desired. The sequence uses a pulse sequel for saturation [6] for each image. The image readout is single shot using parallel imaging acceleration to reduce the imaging duration. The AIF uses a FLASH readout, whereas the higher resolution myocardial images may be either b-SSFP or FLASH, selected by the user. The measurement begins with the acquisition of PD weighted images used for surface coil intensity correction and normalization of signal values. The PD images are acquired using a low flip angle FLASH readout without SR preparation to minimize artifacts of b-SSFP at low flip angle [7]. An optional chemical shift fat suppression may be used to mitigate artifacts due to the presence of fat around the heart. Fat suppression is used in this study.

Saturation efficiency is very important in quantification since the conversion of signal intensities to gadolinium concentration depends on a known signal recovery and independence of the signal from slice to slice that is achieved by resetting the magnetization to zero for each image. With high saturation efficiency it is also possible to prescribe a mixture of long and short axis views without cross-talk between slices due to the readout. The SR preparation using the 6-pulse design [6] was chosen since it had excellent saturation efficiency over a wide range of off-resonance and effective transmitter flip angle (FA) which may vary across the heart. A BIR-4 design [6] achieved excellent saturation performance with a shorter duration but was found empirically to have specific absorption rate (SAR) limitation at higher heart rates particularly at higher field strength such as 3 Tesla. The 6-pulse design consisted of non-selective RF pulses with tailored flip angles separated by gradient spoilers. The voltage of the RF pulses was maximized in order to reduce the pulse duration, and the duration was modulated to achieve the specified FAs. The pulse amplitude corresponded to approx. 27 μT at 1.5 T and was reduced to approx. 11 μT at 3 T to reduce the SAR. To ensure that the sequence would not terminate at run time due to average SAR monitor responding to actual changes in the heart rate, a post-scan acquisition delay of up to 100 s was allowed to increase the averaging interval. The performance of the SR preparation in both blood and myocardium was characterized by simulation for different gadolinium contrast concentrations. At 1.5 T, the SR preparation was 26 ms including 1 ms pre- and 4 ms post-spoiler gradients.

Signal intensities were converted to gadolinium concentration, [Gd], in order to linearize the relationship of signal and [Gd] and to be able to have a common scaling between the AIF and myocardial signals which are acquired using different protocols. Cernicanu, et al. [8] proposed a method for conversion of normalized signal intensities using an analytic expression for readout using a gradient recalled echo (GRE) protocol. This formulation was extended to the dual sequence using a numerical Bloch calculation [9] which permitted application to b-SSFP readout of myocardium and FLASH readout of the AIF.

Proton density weighted images were acquired at the start of the scan for both AIF and myocardial image slices using a FLASH sequence without the SR preparation. The timing of the PD images matched the SR prepared images such that the images were acquired at the same cardiac phase. The images were used to correct the surface coil variation and were used as the signal normalization for look-up table (LUT) linearization. LUT calculations assumed that the native tissue T1 for the PD is prior to contrast (T1_0_), therefore it was important that the PD signal intensity was sufficiently independent of the actual T1 since acquisition of rest perfusion scans typically follow the stress scans after only several minutes at which time the actual T1 is not fully recovered, i.e., the actual gadolinium concentration [Gd] > 0. The dependence of PD signal amplitude versus [Gd] for myocardium and blood tissue was calculated through simulation. This led to a selection of readout FA = 5°. Note that coil sensitivity maps, used both in parallel imaging reconstruction and adaptive coil combination, consisted of the average of all time frames including PD weighted images, therefore the number of PD frames acquired was set equal to the parallel imaging acceleration factor of the myocardial imaging (in this case, *R* = 3). The first PD image was used for normalization to avoid signal loss caused by previous heartbeat images.

### Arterial input function

The AIF was acquired immediately after the R-wave trigger and was selected as the most basal of the slices prescribed in the first slice group. The AIF used the 6-pulse sequel for saturation preparation as described above followed by a dual echo low FA FLASH readout. The protocol parameters are listed in Table 1. A short readout (64 point) with wide bandwidth (3900 Hz/pixel) and short duration RF pulses (250 μs, time-bandwidth product = 2.0) were used to achieve low T2* losses (TE_1_ = 0.76 ms). T2* dephasing loss has been a known concern in estimating AIF and conversion to [Gd] and approaches to this problem have focused either on minimizing the loss by choosing adequately short echo time (TE) [10] or on correcting for T2* loss based on modeling the relationship between T1 and T2* [11, 12]. In this work, the dual sequence approach was modified to incorporate a 2 echo acquisition for measurement of T2* during the bolus passage. A dual echo acquisition with monopolar readout was used to acquire a second echo (TE_2_ = 1.76 μs) which was used for direct estimation of T2* during the first pass. The ratio of the 2 echo signals S1/S2 = S0 exp((T2-T1)/T2*) was used to calculate the signal amplitude S0 without T2* loss. This was performed for a blood pool region signal after left ventricle (LV) blood pool segmentation.

**Table 1:** Protocol parameters for AIF imaging sequence at 1.5 T

	FLASH
TE	0.76 & 1.76 ms
TR	2.45 ms
FA	5°
Matrix	64×34
FOV (typical)	360x270x10 mm^3^
PE order	Linear
Parallel imaging	TPAT2
TI	23.8 ms
SR prep	6-pulse (26 ms incl. spoilers)
Imaging duration	42 ms
Total duration	68.2 ms

Blood pool segmentation was performed on the motion corrected low resolution AIF image series to extract arterial input function intensities signals for both echoes. First, the AIF PD image is used to detect the noise background. Since the noise standard deviation (SD) is unity after the SNR unit reconstruction [13], a simple threshold of 3 SD’s was used. For all foreground pixels as determined by the noise mask, the time intensity curves are analyzed using a scale-space based detector [14]. Pixels with top 10% upslope and AUC values are picked as the candidates for LV blood pool mask. A connected component analysis is then used to separate RV and LV pixels based on the time to peak enhancement. The final LV blood pool mask is calculated using a further erosion step which seeks to drop border pixels which are a mixture of blood and myocardial tissue.

In order to shorten the imaging duration, 2-fold acceleration was achieved using parallel imaging with temporal generalized autocalibrating partially parallel acquisitions (TGRAPPA) [15]. In order to minimize the non-linear response due to saturation at high gadolinium concentration a short saturation delay (TS) is desired, where TS is defined as the time from saturation to the k-space center. There is a tradeoff between the image signal-to-noise ratio (SNR), which is reduced at low TS, and linearity, which is improved at low TS. Early designs used a centric readout ordering to minimize TS [16, 17]. In centric ordering the saturation delay is nearly the trigger delay (TD), which may be as short as the gradient spoiler following the RF saturation (4 ms). The difficulty with centric order is 2-fold: 1) the SNR of the baseline images is too low, and 2) the k-space weighting during the saturation recovery leads to a strong high pass spatial filter that enhances the edge of the blood pool. The high pass spatial filter is problematic for accurate calculation of gadolinium concentration since the effective TS is a function of spatial frequency and will vary depending on how the blood region is segmented. For this reason, a linear acquisition ordering was chosen. Although not perfectly linear at high [Gd], it was sufficiently linear to enable LUT correction.

Using a low resolution image and parallel imaging factor 2, the k-space center was typically at *N* = 9 pulses. The sensitivity of the blood signal to in-flowing spins was estimated by comparing the signal after 9-pulses compared to the signal assuming all spins were new.

Conversion of the signal to gadolinium concentration, [Gd], was performed by LUT based on Bloch signal calculations. In this way, the LUT corrected signal was directly proportional to [Gd] and importantly was in the same units as the LUT corrected myocardial signal which was acquired with a different imaging protocol. The LUT was applied to the normalized signal SR/PD where SR and PD were the saturation recovery and proton density weighted images, respectively. It was important that the normalized signal SR/PD was not strongly dependent on the actual transmitted FA. The sensitivity of the LUT to transmit FA was calculated through simulation.

The readout of the PD image for the AIF may influence the initial magnetization of the 1^st^ myocardial image which is at the same slice location since there is no SR preparation for the PD image. For this reason, a low PD FA is used (5°) which minimizes this effect. After 17 RF pulses (time bandwidth 2.0), the magnetization is reduced approximately 2%, as calculated by Bloch simulation.

### Myocardial Imaging

The 2D multi-slice myocardial imaging sequence used the same 6-pulse sequel saturation preparation, followed by a trigger delay (TD) and single shot readout. The single shot readout was either FLASH or b-SSFP. Protocol parameters at 1.5 T are listed in Table 2 and may vary slightly at 3 T. The readout used parallel imaging with 3-fold acceleration using TGRAPPA, and there were 3 PD frames without SR using a FLASH readout at the start of the sequence. An optional chemical shift fat saturation may be used without any penalty in the timing since the TD accommodated the fat saturation RF pulse. Although the gadolinium concentration in the myocardium is typically < 1 mmol/L, the signal response is still somewhat non-linear and therefore, the normalized signal SR/PD was corrected by a LUT which converts the myocardial signal to gadolinium concentration units, [Gd]. The sensitivity of the LUT correction to the actual transmitted FA was calculated by simulation. The b-SSFP FA was limited to 50° in order to reduce sensitivity of LUT correction to variations in actual transmitted FA and well as to reduce the average SAR.

**Table 2:** Protocol parameters for myocardial perfusion CMR sequence at 1.5 T

	FLASH	SSFP
PD frames	3	
PD FA	5° (FLASH)	
TE	1.0 ms	1.04 ms
TR	2.1 ms	2.5 ms
Bandwidth	1085 Hz/pixel	
FA	14°	50°
Matrix	192×111 (1.9×2.4 mm^2^)	
Partial Fourier	3/4	
Asymmetric echo	weak	
FOV (typical)	360x270x8 mm^3^	
PE order	Linear	
Parallel imaging	TPAT3	
TS/TD	100/62 ms	95/40 ms
SR prep	6-pulse (26 ms including spoilers)	
Fat saturation	optional	
Imaging duration	59 ms	70 ms
Total duration	143 ms/slice	142 ms/slice
3 slices + AIF	497 ms (>120 bpm)	495 ms (>120 bpm)

**Fig. 3:**
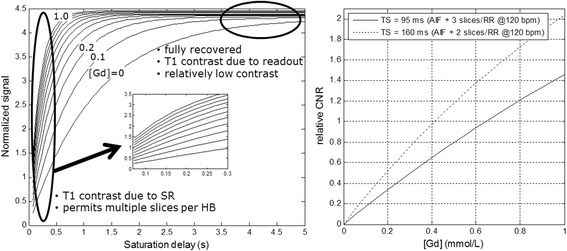
Normalized saturation recovery myocardial signal (SR/PD) for b-SSFP protocol (Table 2) versus saturation delay (TS) for various values of tissue gadolinium concentration, [Gd] (left). With short TS protocols it is possible to acquire multiple slices per heart beat with T1 contrast. The contrast to noise ratio increases with TS, with increasing signal non-linearity and eventually at very long TS there is low contrast as the signal recovers. The CNR vs [Gd] is plotted for 2 values of TS (right) corresponding to T2 = 95 and 160 ms, corresponding to 3 and 2 slices/RR at a heart rate of 120 bpm. The increased TS can achieve approx. 40% higher CNR at the cost of less spatial coverage

The duration of actual signal shot image was 70 ms for SSFP protocol using factor 3 acceleration and Partial Fourier factor of ¾ with the latter part of k-space omitted. There is a trade-off between contrast-to-noise ratio (CNR), spatial coverage (number of slices per RR), and linearity as illustrated in Fig. 3. The protocol was designed to work at a heart rate of 120 bpm which is commonly seen for patients under adenosine stress. It was possible to acquire the AIF plus 3 slices at 120 bpm with the proposed protocol using TS = 95 ms, or AIF plus 2 slices using TI = 160 with increased CNR. It is also possible to prescribe 2x the number of slices by acquiring slices at 2RR intervals. Although there is a gain CNR with longer TS, there is also a loss in performance when using 2RR sampling since there will be fewer samples of the myocardial signal during the first pass measurement.

### Image reconstruction & flow estimation

**Fig. 4:**
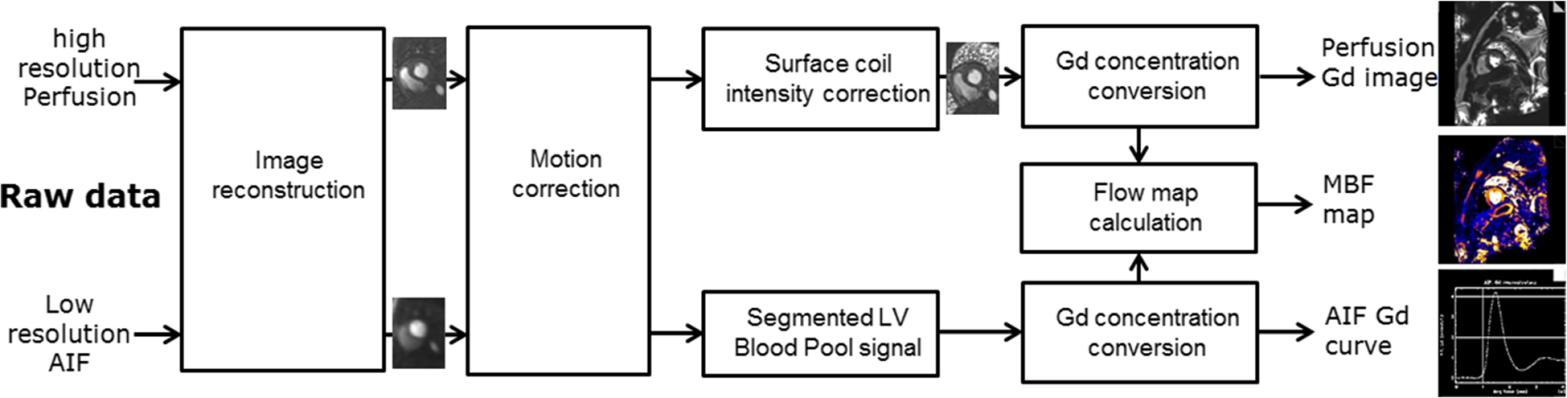
Overview of processing steps. Raw data is reconstructed, respiratory motion corrected, and normalized to correct variation in surface coil intensity. Normalized data are linearized by look-up-table conversion of signal intensities to gadolinium contrast agent concentration, [Gd]. Myocardial blood flow is estimated from the AIF and myocardial pixel time series [Gd] values

Image reconstruction and processing steps are diagrammed in Fig. 4. Parallel imaging was used to accelerate the acquisition of both AIF and myocardial images. This helped to minimize cardiac motion contribution to dark rim artifacts (DRA) [18, 19] that appear as false perfusion defects, and to achieve adequate spatial coverage. Parallel imaging used TGRAPPA reconstruction with coil maps estimated by integrating the complete dataset [15]. All acquisitions were with normal free breathing and parallel imaging auto-calibration was performed on the complete dataset resulted in images free of aliasing artifacts. Individual coil images are adaptively combined to minimize noise bias prior to magnitude detection.

Raw filtering was used to reduce edge ringing [20] (Gibb’s ringing) and to mitigate contamination of the measurements due to fat. In the low resolution AIF, a true Hanning window (i.e., without modification as in Tukey windows) was used to mitigate the influence of fat on the blood pool. For the low resolution AIF images the chest wall fat may be only 10–20 pixels from the LV blood pool. The Hanning window point spread function is <0.02% at a distance greater than 10 pixels whereas an un-windowed reconstruction would be <3.5% at this distance. The loss in resolution at full width half maximum is approx. 60%. In the higher resolution myocardial images, a truncated Gaussian filter was used, truncated to a width of 1.5 standard deviations. The first sidelobe was reduced to 36% compared to unweighted with a mainlobe broadening of 16% at full width half maximum.

Reconstruction and processing were implemented within the Gadgetron software framework [21] and were in-line and fully automatic. All images were respiratory motion corrected using a non-rigid image registration [22]. All images were reconstructed in SNR units [13] to facilitate image scaling, SNR measurements, and calculation of fixed threshold noise masks.

Myocardial blood flow was calculated multiple tissue models: 1) a Fermi model [2], and 2) a blood tissue exchange (BTEX) model originally developed by Bassingthwaighte [23] which is a distributed model described by the partial differential equations (PDE):

1 $$ \frac{\partial {C}_p}{\partial t}=\frac{- FL}{V_p}\cdot \frac{\partial {C}_p}{\partial x}+\frac{PS}{V_p}\cdot \left({C}_{isf}-{C}_p\right)+{D}_p\cdot \frac{\partial^2{C}_p}{\partial {x}^2} $$

2 $$ \frac{\partial {C}_{isf}}{\partial t}=-\frac{PS}{V_{isf}}\cdot \left({C}_{isf}-{C}_p\right)+{D}_{isf}\cdot \frac{\partial^2{C}_{isf}}{\partial {x}^2} $$

where subscripts p and isf correspond to plasma and interstitial fluid, respectively, C the contrast agent concentration, F is the blood flow, PS is the permeability surface area product for the capillaries, V_p_ and V_isf_ are the intracapillary plasma and interstitial fluid volume, respectively, D is the axial diffusion coefficient, L is the capillary length, and x is the distance along the capillary. These equations follow Bassingthwaighte [23] Eqs. (1) and (3) with the term for regional consumption ignored, assuming a single term for capillary leakage dominated by the gaps in the capillary wall, and that the gadolinium based contrast agent is extracellular. The BTEX implementation solved for 4 unknown parameters: myocardial blood flow, interstitial volume, plasma volume, and the permeability surface area product that governs the extraction efficiency, with fixed values for other parameters. The PDE was applied to the AIF to calculate the myocardial response for each set of model parameters, and the parameters with the minimum mean squared error were used as the estimate. The Fermi model was fit to the first pass only and the BTEX model was fit to the entire measurement. The influence of T2* correction of the AIF on myocardial blood flow was analyzed.

### Phantom validations

The sequence was simulated by Bloch equations to calculate the transverse magnetization as a function of all the protocol and tissue parameters in order to construct the LUT corrections for both the AIF and myocardial imaging protocols. Input to the LUT was the normalized signal SR/PD, where PD used the FLASH protocol and SR used either a b-SSFP or FLASH protocol. LUT were validated by phantom measurement by comparing the estimates of [Gd] after LUT correction with the known [Gd] using least squares fitting. A set of gadolinium doped saline phantoms were constructed at concentrations up to 10 mmol/L using both Gadoterate meglumine (Dotarem, Guerbet LLC) and Gadobutrol (Gadavist, Bayer Healthcare). LUT estimates of [Gd] vs known [Gd] were calculated with and without T2* correction. The phantom T1 values were measured using an inversion recovery GRE sequence at multiple inversion times (TI) with TR = 10s such that the longitudinal magnetization was fully relaxed after each RF excitation, and T1 was estimated by 3-parameter fitting to the mono-exponential inversion recovery S = A-Bexp(-TI/T1). The phantom T2 values were measured using a spin echo sequence (TR = 10s) with varying echo times (TE) and using T2 estimates from a 2-parameter fit to the mono-exponential decay curve, S = Aexp(-TE/T2). The coefficients for relaxivity rates (r1 and r2) were calculated from the T1 and T2 measurements vs known [Gd] using linear fitting, i.e., R1 = R1_0_ + r1[Gd] with R1 = 1/T1.

### In-vivo data measurements

The proposed sequence and in-line flow mapping was performed at stress and rest on 29 healthy normal volunteers (11 men and 18 women, mean age 25.4 ± 5.7 years) at the Karolinska University Hospital, Stockholm, Sweden. Studies were approved by the local Ethics Committee. Anonymized data was analyzed at NIH with approval by the NIH Office of Human Subjects Research OHSR (Exemption #13156). All imaging was performed at 1.5 T (Magnetom AERA, Siemens, software version VE11A). Gadolinium (Gd) contrast agent (Gadobutrol) was administered as a bolus with 0.5 dose (0.05 mmol/kg) at 4 mL/s with 20 mL saline flush. One cannula was used for administration of adenosine and another cannula for the administration of contrast agent. Adenosine was administered by continuous infusion for approximately 8 min at a dose of 140 μg/kg/min to allow for additional research scans at stress just prior to contrast administration. The SSFP protocol was used in this study with fat saturation enabled.

In-vivo studies were performed to test the sequence and LUT conversion of signal intensities. Peak [Gd] was measured for the AIF blood pool signal and myocardium, as well as peak SNR in the myocardium from SNR scaled signal intensities. Blood pool T2* values at peak [Gd] were measured as well as the influence of T2* correction on estimates of myocardial blood flow. Duration of the bolus first pass was measured automatically from the AIF signal from the foot of the curve on the upslope of the AIF to the foot of the downslope. The improvement in linearity of the AIF after conversion to gadolinium concentration was measured by the ratio of the AIF peak to valley following the peak, for the raw signal intensities and for the LUT corrected [Gd].

## Results

### Simulations and Look-up table calculations

**Fig. 5:**
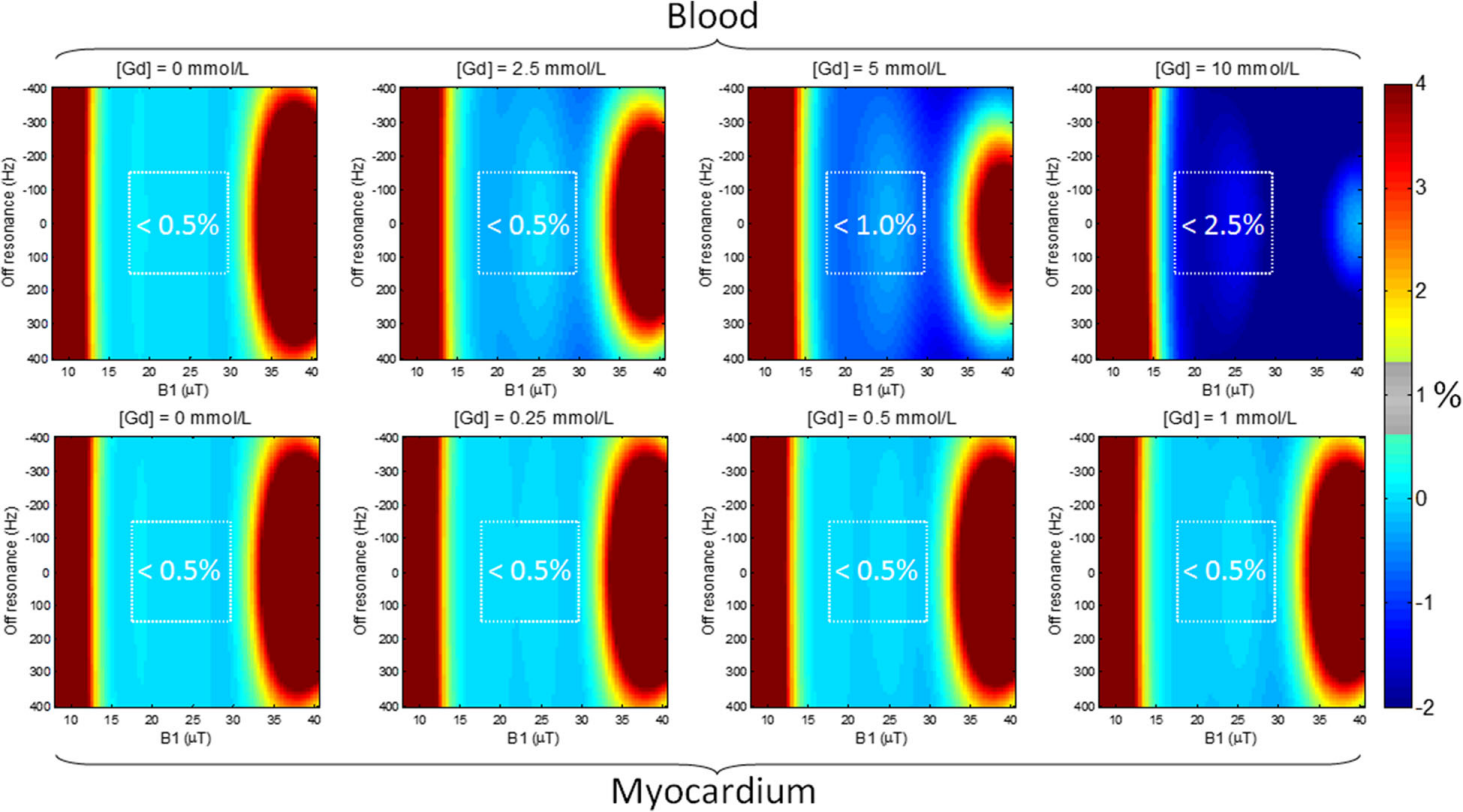
Performance of saturation recovery preparation for blood and myocardium vs off-resonance and transmitter B1 for varying [Gd]. Over the target design range of ±150 Hz and 17.5–29.6 μT (65–110% of effective FA) (dotted white box), the residual magnetization was < 0.5% in the myocardium for [Gd] up to 1 mmol/L, and for blood was < 0.5% up to 2.5 mmol/L, < 1% up to 5 mmol/L, and < 2.5% up to 10 mmol/L

Performance of the 6-pulse saturation recovery preparation is shown in Fig. 5. Over the target design range of ±150 Hz and 17.5–29.6 μT (65–110% of effective FA) (dotted white box), the residual magnetization was < 0.5% in the myocardium for [Gd] up to 1 mmol/L, and for blood was < 0.5% up to 2.5 mmol/L, < 1% up to 5 mmol/L, and < 2.5% up to 10 mmol/L.

**Fig. 6:**
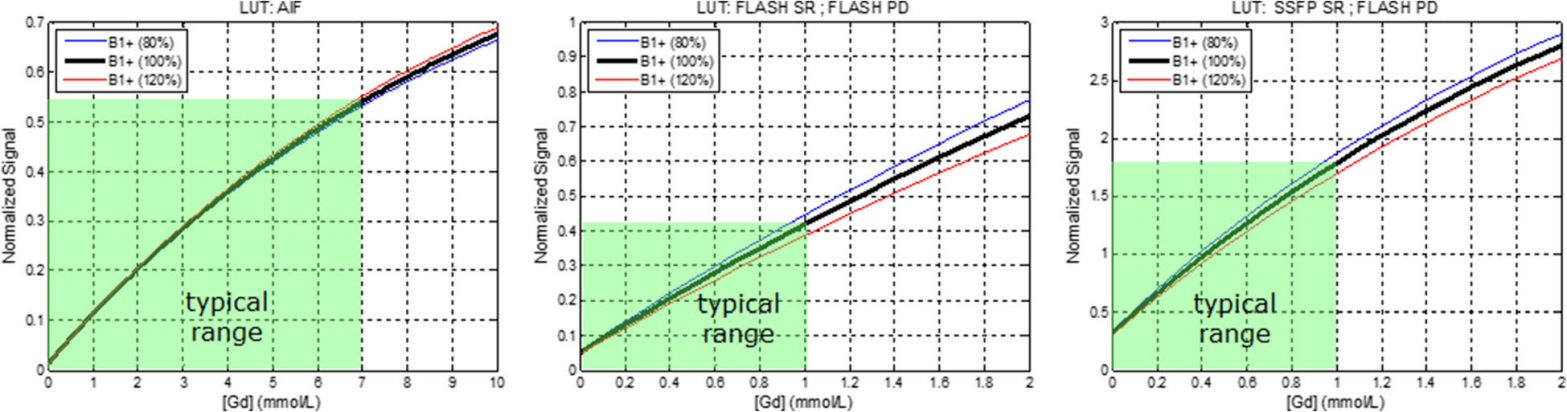
Look-up-tables (LUT) relating the normalized signal (SR/PD) and gadolinium concentration, [Gd], for arterial input function (left), myocardium using SR-FLASH (center), and myocardium using SR-SSFP (right) for protocols in Tables 1 and 2. Typical ranges of [Gd] are highlighted by green shading. Sensitivity to actual transmitted flip angle is indicated by plotting variation of ±20% in B1+ which was <10% at 1 mmol/L in the myocardium, and <3% in the blood up to 10 mmol/L

LUT’s relating the normalized signal (SR/PD) and [Gd] were calculated for ±20% variation in transmitted B1 from nominal (Fig. 6). The error in [Gd] due to the LUT using the assumed specified FA rather than actual was <10% over this range up to 1 mmol/L and <3% in the blood up to 10 mmol/L.

The T1 weighting of the PD images had < 1% variation in signal intensity over 0-2 mmol/L in the myocardium, and <1% over 0-1 mmol/L in the blood. The [Gd] in blood was < 1 mmol/L for a rest study following stress by several minutes, therefore using a fixed value of native T1_0_ had <1% effect on the LUT. The effect of in-flow on the AIF LUT was calculated to be <4% assuming all spins were refreshed every RF pulse.

### Phantom measurements

**Fig. 7:**
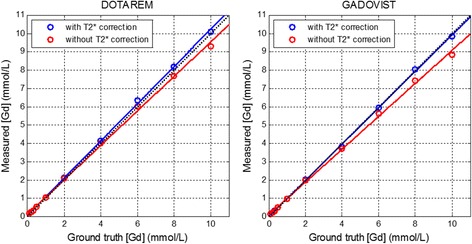
Measured [Gd] vs true [Gd] for phantoms estimated with and without T2* correction for the AIF protocol with the line of identity shown as dotted black line

**Fig. 8:**
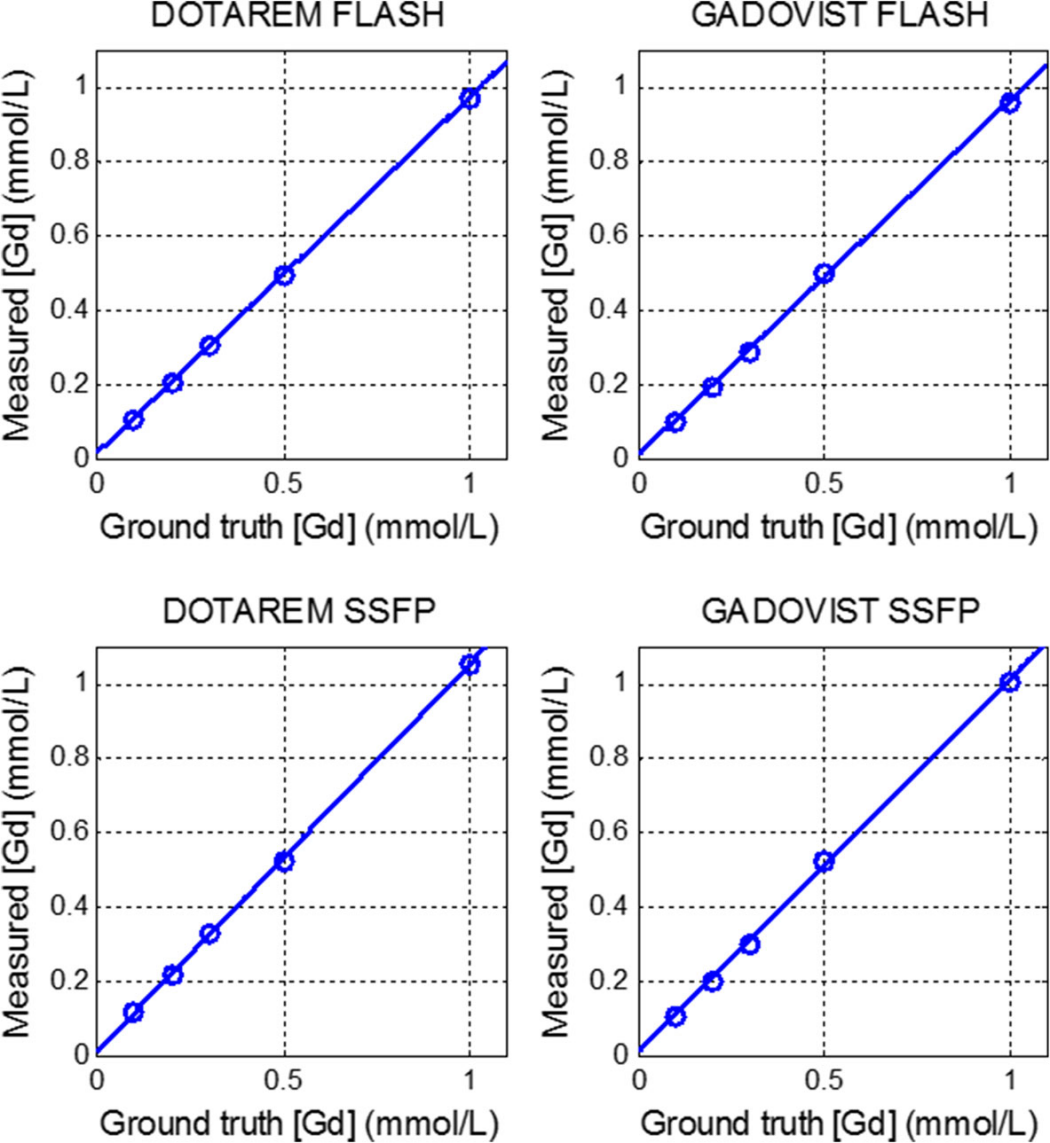
Measured [Gd] versus true [Gd] for phantoms for the FLASH and SSFP myocardial imaging protocols with TS = 95 ms

Measurement of r1 and r2 relaxivities was made for both Gadobutrol (Gadovist^®^) and Gadoterate meglumine (Dotarem^®^) doped saline phantoms. For Gadobutrol, the measured values for r1 and r2 were 5.5 L/mmol/s and 6.8 L/mmol/s, respectively and for Gadoterate meglumine the measured values were 4.6 L/mmol/s and 5.7 L/mmol/s, respectively. The measured [Gd] versus actual [Gd] is shown with and without T2* correction (Fig. 7). The linear fit for Gadobutrol was [Gd]_estimate_ = 0.99 [Gd] + 0.0002 with T2* correction and was [Gd]_estimate_ = 0.90 [Gd] + 0.08, without T2* correction. The linear fit for Gadoterate meglumine was [Gd]_estimate_ = 1.02 [Gd] + 0.07 with T2* correction and was [Gd]_estimate_ = 0.94 [Gd] + 0.12, without T2* correction. Measurements of [Gd] for the myocardial signal protocol are shown in Fig. 8 for TS = 95 ms. For TS = 95 ms the fits were 1.004[Gd] + 0.005 and 1.04[Gd] + 0.01 for SSFP protocol with Gadobutrol and Gadoterate meglumine, respectively, and were 0.96[Gd] + 0.01 and 0.96[Gd] + 0.02 for the FLASH protocol with Gadobutrol and Gadoterate meglumine, respectively. The measurements were made for TS = 65 to 125 ms in steps of 10 ms. For SSFP, the slopes of the fits were within 4% of unity slope for all TS values and both agents and for FLASH were within 5%.

### Invivo AIF data

**Fig. 9:**
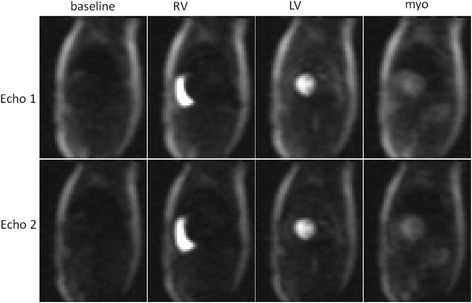
Example AIF images for stress study showing echo 1 and 2 images at baseline and peak enhancement of right ventricle (RV), left ventricle (LV) and myocardium (myo)

**Fig. 10:**
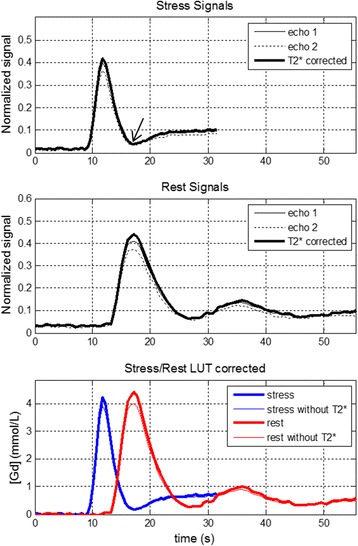
Example of AIF signals for a stress/rest study showing normalized signals (SR/PD) for echo 1 and echo 2 and T2* corrected for stress (top) and rest (middle). The estimated [Gd] (bottom) is shown with and without T2* correction

Adenosine stress studies were conducted on 29 normal healthy volunteers. The peak [Gd] was 3.5 ± 1.2 mmol/L (m ± SD) for stress and 4.4 ± 1.2 mmol/L for rest as measured in the AIF. The T2* in the LV blood pool at peak AIF [Gd] was 10.0 ± 2.4 ms at stress and 9.9 ± 1.7 ms at rest. The duration of the 1^st^ pass was 10.3 ± 2.1 s at stress and 14.7 ± 3.2 s at rest. Example AIF images for echo 1 and echo 2 are shown in Fig. 9 for stress, and normalized AIF signal curves are shown in Fig. 10. In this example, the T2* corrected signal was approximately 8% higher than the echo 1 peak signal, which led to approximately 10% greater [Gd] after LUT correction. For 29 subjects, the peak to valley ratio was 5.6 for the raw signal intensities without correction, and was 8.3 for the LUT corrected AIF in gadolinium concentration units. The valley is indicated by the arrow in Fig. 10 stress signal intensity plot. This represents approximately 48% improvement in linearity.

### Invivo myocardium data

**Fig. 11:**
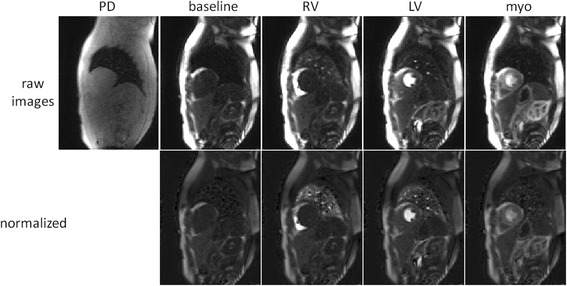
Example of myocardial stress perfusion images for a stress study before (top) and after (bottom) intensity normalization using the PD image

**Fig. 12:**
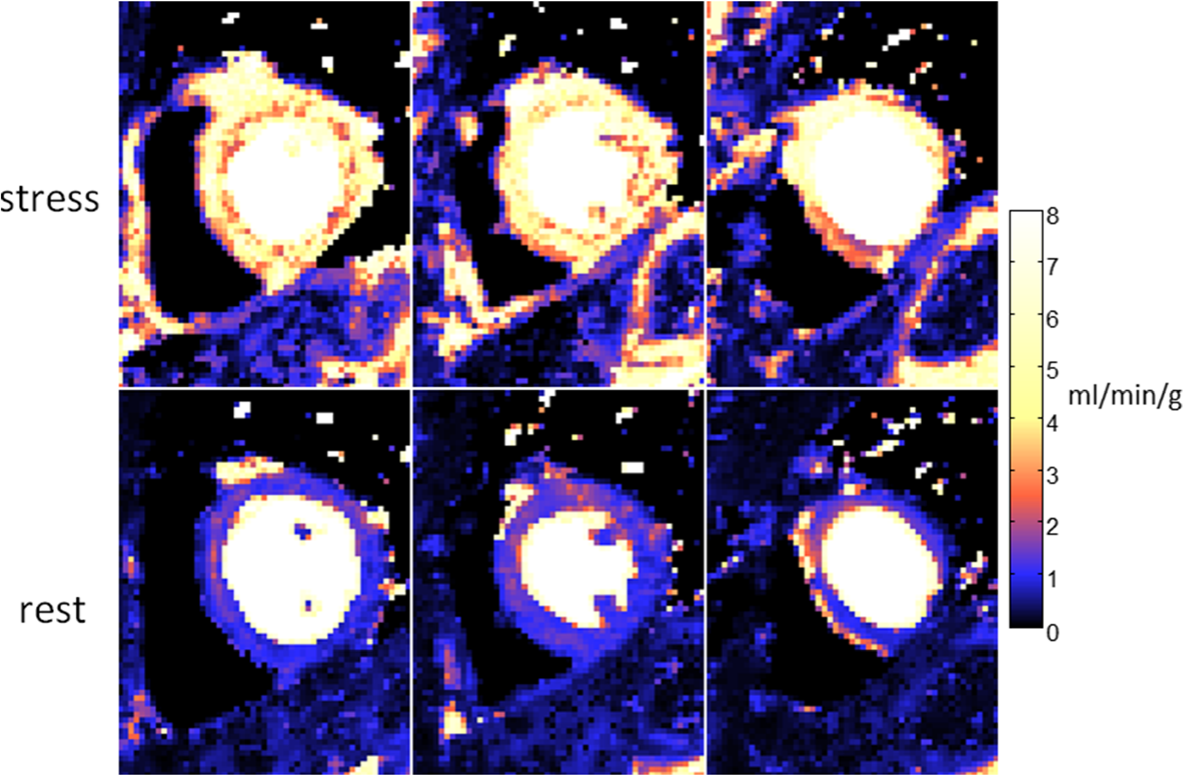
Example of stress (top) and rest (bottom) myocardial blood flow maps for normal subject. Stress flow is appox. 4.1 mmol/min/g and rest flow is approx. 1.2 mmol/min/g

**Fig. 13:**
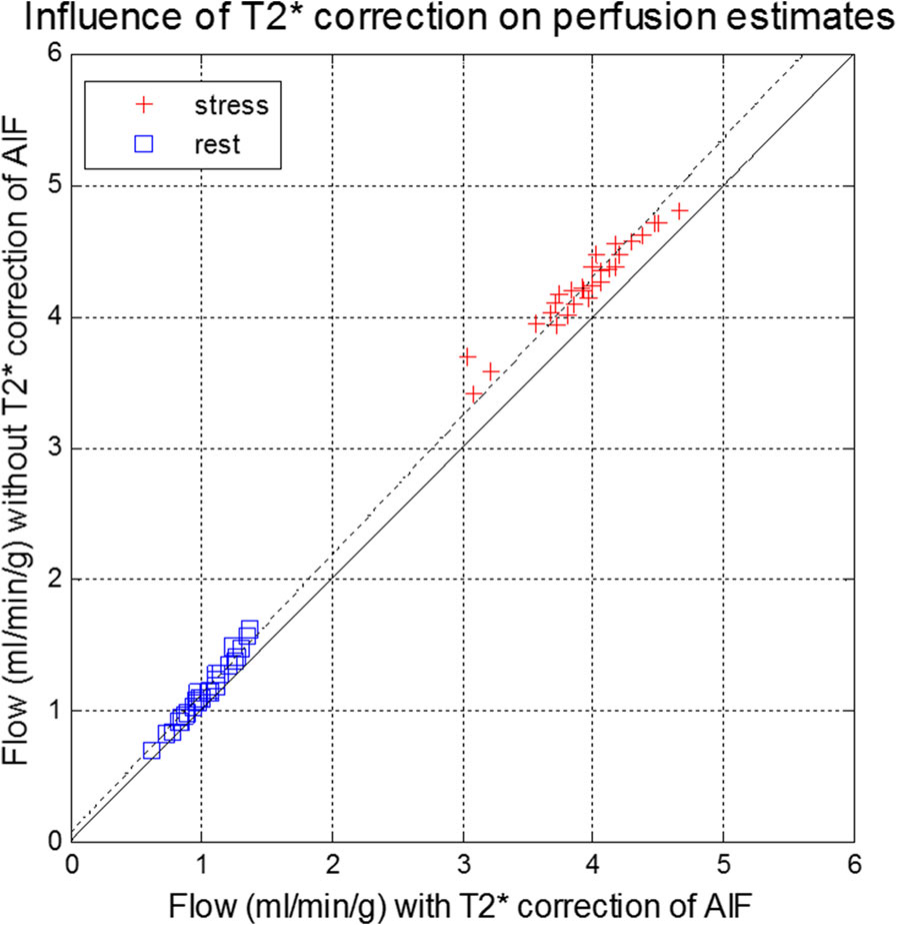
Influence of T2* correction on flow comparing myocardial blood flow estimates with and without T2* correction

The SNR of the myocardial time intensity was measured on a pixel-wise basis using SNR scaled reconstruction and measured at peak myocardial enhancement. Peak myocardial SNR was 21.8 ± 7.6 at stress and the peak [Gd] was 0.49 ± 0.15 mmol/L. Example of myocardial stress perfusion images are shown before and after normalization (Fig. 11) and myocardial blood flow maps are shown in Fig. 12. Images are well saturated as observed at baseline. Influence of T2* correction on flow comparing myocardial blood flow estimates with and without T2* correction is shown in Fig. 13. Without T2* correction the myocardial perfusion estimates of blood flow are overestimated by 10%. Estimates of perfusion stress flow using the BTEX model was 3.93 ± 0.38 and rest flow was 1.03 ± 0.19 ml/min/g (N = 29). Estimates for extraction fraction were 0.5 ± 0.04 and 0.85 ± 0.03, at stress and rest, respectively. Estimates of the permeability surface area product (PS) were 1.55 ± 0.2 and 1.33 ± 0.21 (ml/min/g), at stress and rest, respectively. Estimates for the interstitial volume fraction (%) were 27.4 ± 5.9 and 24.8 ± 5.9, at stress and rest, respectively. Estimates for the blood volume fraction (ml/g) were 13.0 ± 0.85 and 9.2 ± 0.76, at stress and rest, respectively. Estimates of perfusion flow using the Fermi model fit over the 1^st^ pass were 3.4 ± 0.39 and 0.95 ± 0.16 ml/min/g, at stress and rest, respectively.

Raw images for a typical case corresponding to the example in Figs. 10 and 12 are provided as supplemental data as movies to include raw AIF images at stress and rest for both echo times before [see Additional file 1] and after respiratory MOCO [see Additional file 2], and the multislice stress and rest myocardium images including raw images [see Additional file 3], MOCO images [see Additional file 4], and MOCO images including surface coil correction [see Additional file 5].


Additional file 1: Example raw AIF images for both echos at stress (top) and rest (bottom). (5390 kb)
Additional file 2: Example MOCO AIF images for both echos at stress (top) and rest (bottom). (5590 kb)
Additional file 3: Example raw myocardium images at stress (top) and rest (bottom). (4360 kb)
Additional file 4: Example MOCO myocardium images at stress (top) and rest (bottom). (4780 kb)
Additional file 5: Example normalized MOCO myocardium images at stress (top) and rest (bottom). (4780 kb)


## Discussion

The dual sequence approach was chosen for automated, in-line perfusion mapping since it is readily integrated into a clinical workflow. Unlike the dual bolus method, simultaneous measurement of the AIF and myocardial signals avoids physiological variation between bolus injections such as those due to differences in respiration. Another benefit of the dual sequence is that it decouples the measurement of the AIF from the myocardial imaging protocol so that they may be independently optimized. The inherent non-linear response of SR on the AIF was minimized by design of the protocol and post-processing. Earlier dual sequence AIF protocols used centric ordered acquisition to minimize the TS for improved linearity, but this leads to a high pass spatial filtering of the blood pool signal which becomes dependent on gadolinium concentration [10] and creates a dependence on the AIF and how the blood pool is segmented, i.e., the edges of the blood pool will have a longer effective saturation delay. Use of a linear ordering leads to a more homogeneous blood pool image. The saturation delay was minimized by use of parallel imaging acceleration to reduce the number of phase encodes lines actually acquired. The AIF signal with linear phase encoder order was slightly more non-linear than centric but could be corrected by a look-up-table approach based on Bloch signal calculations of the normalized signal (SR/PD). In this way, the blood pool signal could be automatically segmented and the AIF could be reliably estimated. Parallel imaging was used to accelerate the acquisition of myocardial perfusion images in order to reduce the single shot duration and thereby mitigate dark rim artifacts to some extent. Gibb’s ringing was suppressed by raw filtering [24].

### T2* correction

It is difficult to make direct comparison between the T2* values reported here and many of the previously reported measurements since many of the previous publications used different Gd contrast agents (e.g., Magnevist), administered different concentrations of [Gd] (0.05-0.1 mmol/kg), and used different infusion rates (3-7 mL/s). Additionally, several publications measured the signal loss with specific sequence parameters and not actual measurement of T2* with multiple echo times. Thus effects of in-flow due to FA, phase encode acquisition order, slice thickness, will vary between sequences. Finally, not all work was done at 1.5 T. These factors make the direct comparison difficult. The paper by de Bazelaire [15] predicts a value of T2* of 7.5 ms at a peak concentration of [Gd] = 4 mmol at 3 T, and does not give values for 1.5 T which are expected to be longer. By comparison the T2* reported here at 1.5 T for estimated concentration of approx. 4 mmol was approx. 10 ms. Prior work with Magnevist at 1.5 T, 0.1 mmol/kg dose, 5 mmol/s infusion [25] reported T2* values of approx. 9 ms at peak [Gd] concentration. It is also important to adequately shim the volume to minimize intravoxel de-phasing such that the measured value represents the intrinsic T2*.

The correction of T2* in the AIF avoid underestimating the peak [Gd]. Underestimation the input function [Gd] will result in an overestimation of the flow. This is true in general for all perfusion models (BTEX, Fermi, exponential) that are implemented based on the absolute [Gd] signals.

### Conversion to [Gd]

The conversion to gadolinium concentration units facilitated the use of the dual sequence which used a FLASH readout for the AIF and could support either FLASH or SSFP for myocardial perfusion imaging. The relaxivities (r1 and r2) were measured in Gd doped saline phantoms. Although the estimated concentration is dependent on the accuracy of these values, which may be slightly different in blood plasma, the quantified myocardial blood flow has been found through simulation to be quite insensitive since they affect the scale of both blood and myocardium. In-vivo values of blood T2* were significantly lower than for saline phantoms at a given [Gd] as previously reported [26]. The average blood T2* at 1.5 T was approximately 10 ms at 4 mmol/L peak [Gd], whereas the T2* was approximately 25 ms in saline at the same concentration. The T2* decreases with field strength [26] and values as low as 4 ms are likely to be encountered at 3 T using a dose of 0.05 mmol/kg leading to higher signal losses and greater importance for T2* correction. The conversion to [Gd] in the presence of error in FA due to unknown B1 was analyzed through simulation to cause small errors in [Gd] which in turn will result in an error in estimated flow of < 15% for ±20% variation in B1.

### Tissue models

The in-line automated perfusion mapping software has the capability of calculating perfusion estimates based on several different widely used models with differing complexity including the Fermi model [2] and distributed BTEX model [23]. The distributed BTEX model explicitly estimates the permeability surface area (PS) product which is used to calculate an extraction fraction to account for the flow dependent leakage of Gd from the vascular space into the interstitium. The BTEX model has been used in ^13^N-Ammonia PET and validated against microspheres [27]. There is growing interest in distributed tissue models for perfusion [28–30]. A more comprehensive comparison of models will be the subject of a detailed study. Despite significant differences in models, the mean values for perfusion estimates compare reasonably well with other reported values in normal subjects. In a study by Broadbent, et al. [28], perfusion was estimated using both Fermi and a distributed parameter (DP) model. Values for stress and rest perfusion estimates and myocardial flow reserve in that study were 3.8/1.5/2.7 for Fermi and 3.5/1.5/2.5 for DP, as compared to 3.4/0.95/3.6 for Fermi and 3.9/1.03/3.8 for BTEX in the present study. In a study of 10 normal subjects, Hsu, et al. [31] reported values of 3.39/1.02/3.3 using a Fermi tissue model.

### Saturation preparation

The 6-pulse saturation preparation achieved > 99% saturation over a wide range of off-resonance, effective transmitter FA, and gadolinium concentration. Excellent saturation mitigates slice to slice cross-talk and allows the user to prescribe a mixture of short and long axis slices. There is possibility of slight cross talk between intersecting slices for the initial non-saturated PD frames, but this is quite small due to the low FA of PD weighted acquisition protocol. The 6-pulse is designed conservatively, and has recently been reduced to a 5-pulse design to reduce the overall duration and SAR. The newly design preparation has recently been evaluated and perform with >99% saturation over a design range of 50–110% nominal B1+. This newly designed SR preparation is 14.7 ms including crushers which translated to a total AIF duration of 57 ms. Using this 5-pulse design allows for slight increase of the saturation delay from 95 to 105 ms, thereby increasing the SNR while maintaining 3-slices up to 120 bpm.

## Conclusion

A dual sequence for myocardial perfusion CMR and arterial input function measurement has been optimized for quantification of myocardial blood flow. A validation in phantoms was performed to confirm that the signal conversion to gadolinium concentration was linear. The proposed sequence was integrated with a fully automatic in-line solution for pixel-wise mapping of myocardial blood flow and evaluated in adenosine stress and rest studies on *N* = 29 normal healthy subjects. Reliable perfusion mapping was demonstrated and produced estimates with low variability.

## Abbreviations

[Gd]: Gadolinium concentration
AIF: Arterial input function
CMR: Cardiovascular magnetic resonance
CNR: Contrast-to-noise ratio
DRA: Dark rim artifact
FA: Flip angle
FLASH: Fast low angle shot
LGE: Late gadolinium enhancement
LUT: Look-up table
MOCO: Motion correction
PD: Proton density
RF: Radio frequency
SAR: Specific absorption ratio
SR: Saturation recovery
SSFP: Steady state free precession
TD: Trigger delay
TS: Saturation delay
